# On alternative wavelet reconstruction formula: a case study of approximate wavelets

**DOI:** 10.1098/rsos.140124

**Published:** 2014-10-08

**Authors:** Elena A. Lebedeva, Eugene B. Postnikov

**Affiliations:** 1 Mathematics and Mechanics Faculty, Saint Petersburg State University, Universitetsky prospekt 28, Peterhof, Saint Petersburg 198504, Russia; 2 Institute of Applied Mathematics and Mechanics, Saint Petersburg State Polytechnical University, Polytechnicheskay 29, Saint Petersburg 195251, Russia; 3 Department of Theoretical Physics, Kursk State University, Radishcheva st. 33, Kursk 305000, Russia

**Keywords:** wavelet transform, Gaussian function, Morlet wavelet, reconstruction formula, admissibility condition, Hilbert transform

## Abstract

The application of the continuous wavelet transform to the study of a wide class of physical processes with oscillatory dynamics is restricted by large central frequencies owing to the admissibility condition. We propose an alternative reconstruction formula for the continuous wavelet transform, which is applicable even if the admissibility condition is violated. The case of the transform with the standard reduced Morlet wavelet, which is an important example of such analysing functions, is discussed.

## 2. Introduction

The continuous wavelet transform (CWT) on $L_{2}(R)$ is defined as

2.1 $$W_{n,\psi }f(a,b)=\int_{R}f(x)\bar{\psi_{n,a,b}(x)}\;dx,$$

where *n*=1 or *n*=2,

$$\psi_{1,a,b}(x)=\frac{1}{|a|}\psi \left(\frac{x-b}{a}\right)\;\mathrm{and}\;\psi_{2,a,b}(x)=\frac{1}{|a{|}^{1/2}}\psi \left(\frac{x-b}{a}\right),$$

a,b∈R, *a*≠0. For *n*=1, we suppose $\psi \in L_{1}(R)\cap L_{2}(R)$ and $\int_{R}|\psi (x)|\;dx=1$, then the amplitude norm $\int_{R}|\psi_{1,a,b}(x)|\;dx=1.$ For *n*=2, we suppose $\psi \in L_{2}(R)$ and $\int_{R}|\psi (x){|}^{2}\;dx=1$, then the energy norm $(\int_{R}|\psi_{2,a,b}{\left(x){|}^{2}\;dx\right)}^{1/2}=1.$

The CWT is one of the powerful modern analysis tools in various branches of science connected with the processing of non-stationary signals because it allows one to obtain a detailed localized time–frequency decomposition of non-stationary signals ([1] and references therein).

At the same time, the CWT can be applied not only for an analysis and for the decomposition of signals, but also for their reconstruction using time-localized oscillating components. In particular, this framework finds actual implementations in the modern problems of quantum mechanics, because wavelets provide a natural way to represent coherent states corresponding to the mentioned wavelet-based reconstruction [2,3], and neurodynamics [4], where wavelet-like spikes are typical elements of detected activity.

The opportunity for wavelet reconstruction is provided by the admissibility condition [5]

$$C_{\psi }=\int_{R}\frac{|\hat{\psi }(\omega ){|}^{2}}{|\omega |}\;d\omega <\infty$$

that is equivalent to $\hat{\psi }(0)=0$ under the additional assumption $(1+|\cdot {|}^{\alpha })\psi \in L_{1}(R),$ *α*>0. As usual, we denote by $\hat{f}$ the Fourier transform of *f*:  $\hat{f}(\omega )=\int_{R}f(x)\;e^{i\omega x}\;dx.$

Under the admissibility condition, the reconstruction formula takes place for all $f\in L_{2}(R)$

2.2 $$f(x)=\frac{1}{C_{\psi }}\int_{R}\int_{R}\psi_{2,a,b}(x)W_{2,\psi }f(a,\;b)\frac{da\;db}{a^{2}},$$

where the equality is understood in a weak sense. If, in addition, *f* is continuous at x∈R, then (see [6], theorem 3.10) the equality holds at the point x∈R. The analogous result takes place for a pair of different wavelets *ψ*,*g* used for the analysis (the function *ψ*) and the reconstruction (the function *g*). In this case, the admissibility condition consists of two parts: $\omega^{-1}\bar{\hat{\psi }(\omega )}\hat{g}(\omega )\in L_{1}(R),$ and $C_{\psi ,g}:=\int_{R}|\omega {|}^{-1}\bar{\hat{\psi }(\omega )}\hat{g}(\omega )\;d\omega \neq 0.$ And the result itself is read as follows (see [5], proposition 2.4.2): if *ψ*, *g*, $xg(x)\in L_{1}(R),$ $g^{\prime }\in L_{2}(R)$, $\hat{\psi }(0)=\hat{g}(0)=0,$ $f\in L_{2}(R)$ is bounded, *f* is continuous at x∈R, then we obtain

2.3 $$f(x)=\frac{1}{C_{\psi ,g}}\lim_{A_{1}\to 0,A_{2}\to \infty }\int_{A_{1}\leq |a|\leq A_{2}}\frac{da}{a^{2}}\int_{R}g_{2,a,b}(x)W_{2,\psi }f(a,b)\;db.$$

The function can also be reconstructed (see [7, theorem 2.2]) as

2.4 $$f(x)=\lim_{\rho \to \infty \varepsilon \to 0}\int_{\varepsilon }^{\rho }\frac{da}{a}\int_{R}g_{1,a,b}(x)W_{1,\psi }f(a,b)\;db$$

under the following restrictions on the function *f*, and the wavelets *ψ*, *g*:

1. *f* is continuous at x∈R,
2. $\lim_{u\to \infty }(1/2u)\int_{t-u}^{t+u}f(x)\;dx=0$ uniformly in *t*;
3. $\log(2+|\cdot |)\psi \in L_{1}(R),$ and $\log(2+|\cdot |)g\in L_{1}(R),$
4. $\omega^{-1}\hat{\psi }(\omega )\bar{\hat{g}(\omega )}\in L_{1}(R),$
5. $\int_{0}^{\infty }\omega^{-1}\hat{\psi }(\omega )\bar{\hat{g}(\omega )}\;d\omega =\int_{-\infty }^{0}|\omega {|}^{-1}\hat{\psi }(\omega )\bar{\hat{g}(\omega )}\;d\omega =1.$

However, item (5) is more restrictive than the conditions *C*_*ψ*,*g*_≠0, and $C_{\psi }<\infty$ (*ψ*=*g*). The latter condition coincides with (4), and it is the unique admissibility condition for the case of the same decomposition/reconstruction wavelet (formula (2.2)).

Note that the a.e. and $L_{p}(R)$-norm (1≤p<∞) convergence of the inversion formulae is studied in detail in [8–10].

At the same time, even one of the most popular and useful in applications wavelets, the standard reduced Morlet wavelet $\psi_{M}(\xi )=\exp(i\omega_{0})\exp(-\xi^{2}/2),$ does not satisfy the admissibility condition because ${\hat{\psi }}_{M}(0)=C_{1}\exp(-\omega_{0}^{2}/2)$. However, this quantity is sufficiently small for the practically used central frequencies (usually *ω*_0_≥5) that allows one to apply it widely to the signal decompositions, when an exact reconstruction is not necessary [11–14]. Note that the standard reduced Morlet wavelet can be improved using the correction term. As a result, one obtains the exact Morlet wavelet $\psi_{M,\mathrm{ex}}(\xi )=C(\exp(i\omega_{0}\xi )-\exp(-\omega_{0}^{2}/2))\exp(-\xi^{2}/2),$ where *C* is a normalization factor. This one satisfies the admissibility condition. However, the exact Morlet wavelet corresponds to a more complicated interconnection between the frequencies of signal harmonic components and wavelet modulus maxima than the standard reduced Morlet wavelet. For this reason, the reduced version is used more widely in applications as a standard wavelet.

We do not discuss the deeply celebrated background of the classical wavelet reconstruction formula such as the Calderon reproducing formula, Hilbert spaces with reproducing kernels, irreducible unitary representations of Lie groups. Our goal is to prove that the violation of the admissibility conditions actually does not forbid an existence of the exact wavelet inversion. As a result, we suggest an alternative wavelet reconstruction formula that does not require the admissibility condition. As an example, we consider the standard Morlet wavelet for arbitrary central frequencies including the limiting case *ω*_0_→0 when the continuous wavelet transform reduces to the result of diffusion smoothing of a processed signal. We use the Gaussian function to show the difference between the suggested formula and the classical one.

## 3. An alternative reconstruction formula

**Theorem 3.1**

*If f, ψ,* $\omega \hat{\psi }(\omega )\in L_{2}(R)$ *and* $\hat{f},$ $\hat{\psi }\in L_{1}(R),$ *then a.e. on* R

3.1 $$\begin{array}{rl} \frac{1}{\pi }\;v.p.\;\int_{R}\frac{db}{b-x}\int_{R}\frac{\partial }{\partial b}W_{1,\psi }f(a,b)\;da & =\bar{\psi (0)}\;f(x)\\ \text{and}\;\frac{1}{\pi }\;v.p.\;\int_{R}\frac{db}{b-x}\int_{R}\sqrt{|a|}\frac{\partial }{\partial b}W_{2,\psi }f(a,b)\;da & =\bar{\psi (0)}f(x). \end{array}\}$$

*In addition, if* $\mathrm{supp}\;\hat{f}\subset [0,\infty ),$ *then a.e. on* R

3.2 $$-i\int_{R}\frac{\partial }{\partial b}W_{1,\psi }f(a,b)\;da=\bar{\psi (0)}f(b),\;-i\int_{R}\sqrt{|a|}\frac{\partial }{\partial b}W_{2,\psi }f(a,b)\;da=\bar{\psi (0)}f(b).$$

**Proof.**

We consider the case of the amplitude norm. The case of the energy norm can be proved analogously. It follows from the definition of CWT that

$$W_{1,\psi }f(a,\;b)=\int_{R}f(x)\bar{\psi_{a,b}(x)}\;dx=\frac{1}{2\pi }\int_{R}\hat{f}(\omega )\bar{\hat{\psi }(a\omega )}\;e^{i\omega b}\;d\omega .$$

Because $\int_{R}|\hat{f}(\omega )\bar{\hat{\psi }(a\omega )}\omega |\;d\omega <\infty$ for any fixed a∈R, we obtain

$$-i\frac{\partial }{\partial b}W_{1,\psi }f(a,b)=\frac{1}{2\pi }\int_{R}\hat{f}(\omega )\bar{\hat{\psi }(a\omega )}\omega \;e^{i\omega b}\;d\omega .$$

Using $\hat{f},$ $\hat{\psi }\in L_{1}(R),$ we obtain

$$\int_{R}d\omega \int_{R}|\hat{f}(\omega )\bar{\hat{\psi }(a\omega )}\omega |\;da=\int_{R}|\hat{f}(\omega )|\;d\omega \int_{R}|\hat{\psi }(\xi )|\;d\xi <\infty .$$

Whence by the Fubini theorem, we finally obtain

$$\begin{array}{rl} -\int_{R}\frac{\partial }{\partial b}W_{1,\psi }f(a,b)\;da & =\frac{-i}{2\pi }\int_{R}\hat{f}(\omega )\;e^{i\omega b}\;d\omega \int_{R}\bar{\hat{\psi }(a\omega )}\omega \;da\\ & =\frac{\bar{\psi (0)}}{2\pi }\int_{R}(-i)\;\mathrm{sgn}\;(\omega )\hat{f}(\omega )\;e^{i\omega b}\;d\omega \\ & =\frac{\bar{\psi (0)}}{2\pi }\int_{R}\hat{Hf}(\omega )\;e^{i\omega b}\;d\omega =\bar{\psi (0)}Hf(b), \end{array}$$

where *H* is the Hilbert transform. It is an well known that the Hilbert transform is an invertible operator on $L_{2}(R),$ *H*^−1^=−*H*, and the inversion formula *H*^−1^(*Hf*)(*x*)=*f*(*x*) holds true almost everywhere on R. Therefore, (3.1) is proved.

If in addition $\mathrm{supp}\;\hat{f}\subset [0,\infty ),$ then the last chain of equalities is rewritten as follows:

$$-i\int_{R}\frac{\partial }{\partial b}W_{1,\psi }f(a,b)\;da=\frac{\bar{\psi (0)}}{2\pi }\int_{R}\hat{f}(\omega )\;e^{i\omega b}\;d\omega =\bar{\psi (0)}f(b).$$

Therefore, (3.2) is proved. ▪

To illustrate the result of theorem 3.1, we consider reconstruction formula (3.2), choose *n*=1, and suppose *ψ*, *f*∈*S*, where *S* is the Schwartz space. Then, $\hat{f}(\cdot )\bar{\hat{\psi }(a\cdot )}\in S$, therefore *W*_1,*ψ*_*f*(*a*,⋅)∈*S* for any fixed a∈R, *a*≠0. Thus, the derivative (∂/∂*b*)*W*_1,*ψ*_*f*(*a*,*b*) is represented using the derivative of the Dirac delta function *δ*

$$\frac{\partial }{\partial b}W_{1,\psi }f(a,b)=-\int_{R}W_{1,\psi }f(a,t)\delta^{\prime }(t-b)\;dt.$$

One can identify *δ*′(*t*−*b*) and *δ*′_1,*a*,*b*_(*t*). Indeed, for any *ϕ*∈*S*, we obtain

$$(\delta_{1,a,b},\phi )=\int_{R}\delta_{1,a,b}(x)\phi (x)\;dx=\int_{R}\delta (x)\phi (ax+b)\;dx=\phi (b)=(\delta (\cdot -b),\phi ),$$

therefore,

$$(\delta_{1,a,b}^{\prime },\phi )=-(\delta_{1,a,b},\phi^{\prime })=-(\delta (\cdot -b),\phi^{\prime })=(\delta^{\prime }(\cdot -b),\phi ).$$

Thus, if $\mathrm{supp}\;\hat{f}\subset [0,\infty ),$ then we obtain ‘quasi-classical’ form for the reconstruction formula (3.2)

3.3 $$f(x)=\frac{i}{\bar{\psi (0)}}\int_{R}\int_{R}W_{1,\psi }f(a,b)\delta^{\prime }(b-x)\;db\;da.$$

However, (3.3) is not reduced to the known reconstruction results (2.2)–(2.4). In fact, admissibility conditions are violated under the choice *ψ*(*x*)=*C* *e*^−*cx*^2^^ and *g*=*δ*′. Indeed, ${\hat{\delta }}^{\prime }(\omega )=i\omega$ and $\hat{\psi }(\omega )=C_{1}\;e^{-c_{1}\omega^{2}}$, *c*_1_>0

$$C_{\psi ,g}=\int_{R}|\omega {|}^{-1}\bar{\hat{\psi }(\omega )}\hat{g}(\omega )\;d\omega =iC_{1}\int_{R}\mathrm{sgn}(\omega )\;e^{-c_{1}\omega^{2}}\;d\omega =0.$$

The condition (5) cited in the Introduction is also not fulfilled

$$\int_{0}^{\infty }\omega^{-1}\hat{\psi }(\omega )\bar{\hat{g}(\omega )}\;d\omega =-\int_{-\infty }^{0}|\omega {|}^{-1}\hat{\psi }(\omega )\bar{\hat{g}(\omega )}\;d\omega \left(=iC_{1}\int_{0}^{\infty }e^{-c_{1}\omega^{2}}\;d\omega \right).$$

On the other hand, consider the standard reduced Morlet wavelet with the central frequency *ω*_0_, that is $\psi_{M}(x)=C\exp(i\omega_{0}x)\exp(-x^{2}/2)$, where *C* is a factor depending on the chosen norm, and ${\hat{\psi }}_{M}(\omega )=C_{1}\exp(-{\left(\omega -\omega_{0}\right)}^{2}/2).$ It is clear that $C_{\psi_{M}}=\infty ,$ and the item (5) is not fulfilled (set here *g*=*ψ*=*ψ*_M_). So, the reconstruction formulae (2.2) and (2.4) are not applicable. However, *ψ*_M_ satisfies all conditions of theorem 3.1, so one can restore a function *f* by (3.1) and (3.2). This fact can be interpreted by means of the ‘quasi-classical’ formula (3.3). It is sufficient to set formally *ψ*_M_ as the analysing wavelet and *δ*′ as the reconstruction wavelet. In this case, $C_{\psi_{M},\delta^{\prime }}<\infty ,$ *C*_*ψ*_M_,*δ*′_≠0.

## 4. Discussion of possible applications

As has been noted in the Introduction, there was a contradiction between strictly proven known mathematical requirements for the inversion of the transform (the admissibility condition) and practically used wavelets (e.g. the standard reduced Morlet wavelet), which satisfy this condition only approximately. In this work, we have shown that this violation does not prevent the exact invertibility of the wavelet transform and, consequently, non-admissible wavelets can be used for an exact, not approximate, reconstruction.

The standard reduced Morlet wavelet with a small central frequency contains only a small number of oscillations inside of the Gaussian envelope. Therefore, it is best adjusted to the extraction of temporal dynamics of emergence and moving localization for short-time pulses, e.g. of acoustic echo [12] or spike trains in neuroscience [4].

The variation of the central frequency of the Morlet wavelet, which tends to decreasing *ω*_0_ allows one not only to more fully characterize damping properties of oscillations [11], but also to reveal topological properties of attractors in the theory of phase synchronization of chaotic oscillators [13,14].

Moreover, the sufficiently non-restrictive conditions for the function *ψ* used in theorem 2.1 indicate that this method of reconstruction is not limited by the wavelets (in a general sense) but may be also applied to the appropriate non-oscillating analysing kernel functions, for example, to those Gaussian-based, which emerge in the diffusion signal and image time(space)–frequency smoothing and processing [15].
